# RasGAP and Crk bind *Anaplasma phagocytophilum* effector AteA at tyrosine-phosphorylated EPLYA motifs

**DOI:** 10.1128/mbio.02165-26

**Published:** 2026-10-26

**Authors:** Brittany Genera, Eliciane Cevolani Mattos, Prabhat K. Talukdar, EricaRose Warwick, Kelly A. Brayton, Jason M. Park

**Affiliations:** 1 Program in Vector-borne Disease, Department of Veterinary Microbiology and Pathology, Washington State University 6760, Pullman, Washington, USA; University of California, Berkeley, Berkeley, California, USA

**Keywords:** tick-specific effector, rickettsial pathogen, EPIYA motifs, SH2-domain containing proteins, tyrosine kinases, Src kinase, Abl kinase, vector-pathogen interactions

## Abstract

Host cell signaling networks play critical roles in responding to bacterial infections. Intracellular pathogens manipulate these signals to establish a replicative niche in the host cell. Vector-borne rickettsial pathogens, such as *Anaplasma phagocytophilum*, encounter the dual challenge of interfacing with cell signaling in both the mammalian host and the tick vector *Ixodes scapularis*. A central feature that enables *A. phagocytophilum* survival in both mammals and ticks is the type 4 secretion system (T4SS), which secretes effector proteins into the host cell to manipulate biological processes. Only a few *A. phagocytophilum* T4SS effectors have been experimentally tested, primarily in mammalian cells. While AteA has been identified as the first T4SS effector known to be essential for *A. phagocytophilum* survival in the tick, its molecular targets in the tick remained unknown. Here, we investigated AteA interactions with tick cell proteins and a possible mechanism of AteA activation. AteA contains multiple EPIYA-like short linear motifs with the consensus sequence EPLYA. Other known bacterial EPIYA motif effectors, including AnkA from *A. phagocytophilum*, target mammalian phosphotyrosine signaling proteins. We found that AteA is tyrosine-phosphorylated at EPLYA motifs by the non-receptor tyrosine kinases Src and Abl in eukaryotic cells and during tick infection. Protein pull-down assays revealed that *Ixodes* SH2 domain-containing proteins, RasGAP and Crk, preferentially interact with phosphorylated AteA EPLYA motifs. RasGAP and Crk have interconnected roles in cell signaling, suggesting that *A. phagocytophilum* targets these pathways during infection of the arthropod vector.

**IMPORTANCE:**

Tick-borne diseases are on the rise globally, linked to changes in climate, land use, and human-animal interactions. Interrupting vector-borne transmission requires understanding how the vector and pathogens interact. Recent studies have shown ticks detect and respond to pathogens through cellular immune and stress signaling pathways. We previously demonstrated that the tick-borne bacterium *Anaplasma phagocytophilum* encodes a tick-specific secreted effector, AteA, that is essential for the pathogen’s survival in the tick. Here, we showed that AteA is phosphorylated by tick cell kinases, which promotes binding to host cell SH2 domain-containing proteins that are integral to cellular signaling networks. Understanding how these interactions affect signaling pathways in the tick will be essential if interventions targeting the vector are to be developed.

## INTRODUCTION

Rickettsial pathogens make up a diverse group of obligate intracellular bacteria within the order Rickettsiales, encompassing genera such as *Rickettsia*, *Ehrlichia*, *Orientia*, and *Anaplasma* (1, 2). These organisms are transmitted to mammalian hosts through the bites of arthropods, including ticks, fleas, lice, and mites (1, 3). Transmission depends on the ability to establish infection and persist within both arthropod vectors and mammalian hosts, requiring specialized mechanisms for successful colonization, persistence, and dissemination (4–7). A structure central to all rickettsial pathogens is the type 4 secretion system (T4SS), which delivers effector molecules into host cells to mediate host-pathogen interactions (8–11). To date, T4SS effectors have primarily been examined in the context of mammalian infection (12–21); however, we previously demonstrated that the rickettsial pathogen *Anaplasma phagocytophilum* secretes a T4SS effector (AteA) specific to arthropod infection (22).

*Anaplasma phagocytophilum* is the causative agent of human anaplasmosis, the most prevalent rickettsial disease in North America, which is transmitted by the tick vectors *Ixodes scapularis* and *I. pacificus* (3, 23). Like other multi-host intracellular pathogens, *A. phagocytophilum* has evolved a specialized lifecycle that alternates between mammalian and arthropod hosts. In mammalian hosts, *A. phagocytophilum* primarily infects neutrophils (24, 25), while within the tick vector, the pathogen initially colonizes midgut epithelial cells during acquisition and subsequently invades the salivary glands to prepare for transmission to a new host during a blood meal (26, 27). In all cell types, *A. phagocytophilum* subverts immune defenses, inhibits apoptosis, remodels the cytoskeleton, alters host signaling pathways, and redirects vesicle trafficking to establish a protective intracellular niche (28–36). Central to the bacterium’s ability to interact with and remodel host cells across these divergent environments is its repertoire of T4SS effectors (5, 12–19, 22). Transcriptomic analyses have shown that over 40% of *A. phagocytophilum* genes are differentially expressed between mammalian and tick cells, with the bacterium upregulating genes that facilitate survival within the tick environment, including several candidate T4SS effectors with tick-specific expression (37–39). Genetic manipulation of rickettsia is a documented challenge due to their obligate intracellular nature (40), which has slowed progress on effector characterization. Characterization of tick-specifically expressed effectors has lagged further behind that of effectors functioning in the mammalian host due to fewer tools available in the tick system.

To date, at least seven T4SS effectors from *A. phagocytophilum* have been identified, with the first studied effectors being AnkA (APH_0740) and Ats-1 (APH_0859), which are key effectors of mammalian infection. In the cytosol, AnkA is modified by host kinases Src and Abl and interacts with host cell signaling proteins SHP1 and Abi-1 (12, 13). AnkA also enters the neutrophil nucleus, where it changes host gene expression and inhibits immune signaling for bacterial survival (14, 15). Ats-1 targets the mitochondria to block apoptosis, ensuring a stable environment for bacterial replication (16). Several additional effectors have been reported, including HGE-14 (APH_0455), which is highly expressed in mammalian conditions and is translocated to the host nucleus (18); EgeA (APH_0874), which binds to two (mammalian) host proteins at ER–Golgi exit sites and redirects the cargo to facilitate *A. phagocytophilum* survival and proliferation (17); AFAP (APH_0177), an actin filament-associated protein that was found to interact with the host nucleoli and inhibit apoptosis (19); and APH_0215, which was reported to interact with the trans-Golgi (41). AmpA, APH_0032, and APH_906 have been studied as effectors but may be translocated in a T4SS-independent manner (42–44). Additionally, we previously reported AteA, the first tick-specific *A. phagocytophilum* T4SS effector. AteA is exclusively expressed during tick cell infection, is essential for growth in tick cells, and is important for colonization of the tick from a blood meal. Ectopically expressed AteA localizes tightly to the host cell’s actin cortex (22). However, the molecular binding targets of AteA in tick cells have yet to be defined.

Bacterial effectors mimic host proteins by encoding short linear motifs (SLiMs), short stretches of amino acids that mediate specific protein-protein interactions or post-translational modifications (45). AteA was found to contain several instances of a SLiM resembling the effector motif EPI/LYA. EPI/LYA motifs have been characterized in critical effectors from a small but diverse range of bacterial pathogens (46, 47), including AnkA from *A. phagocytophilum* (13). When phosphorylated by host tyrosine kinases, these motifs become high-affinity binding sites for the SH2 domains of host signaling proteins (46, 48). These interactions allow the bacteria to manipulate host cell signaling, controlling a wide range of cellular functions (46, 49), including actin cytoskeletal rearrangements (50–56). In the present study, we show that AteA EPIYA-like motifs are phosphorylated by Src and Abl family kinases and recruit SH2 domain-containing host proteins, including the *I. scapularis* Crk signaling adaptor protein and the GTPase-activating protein RasGAP, suggesting that AteA uses these motifs to engage host signaling pathways in the tick.

## RESULTS

### AteA contains EPIYA-like motifs

**Fig 1:**
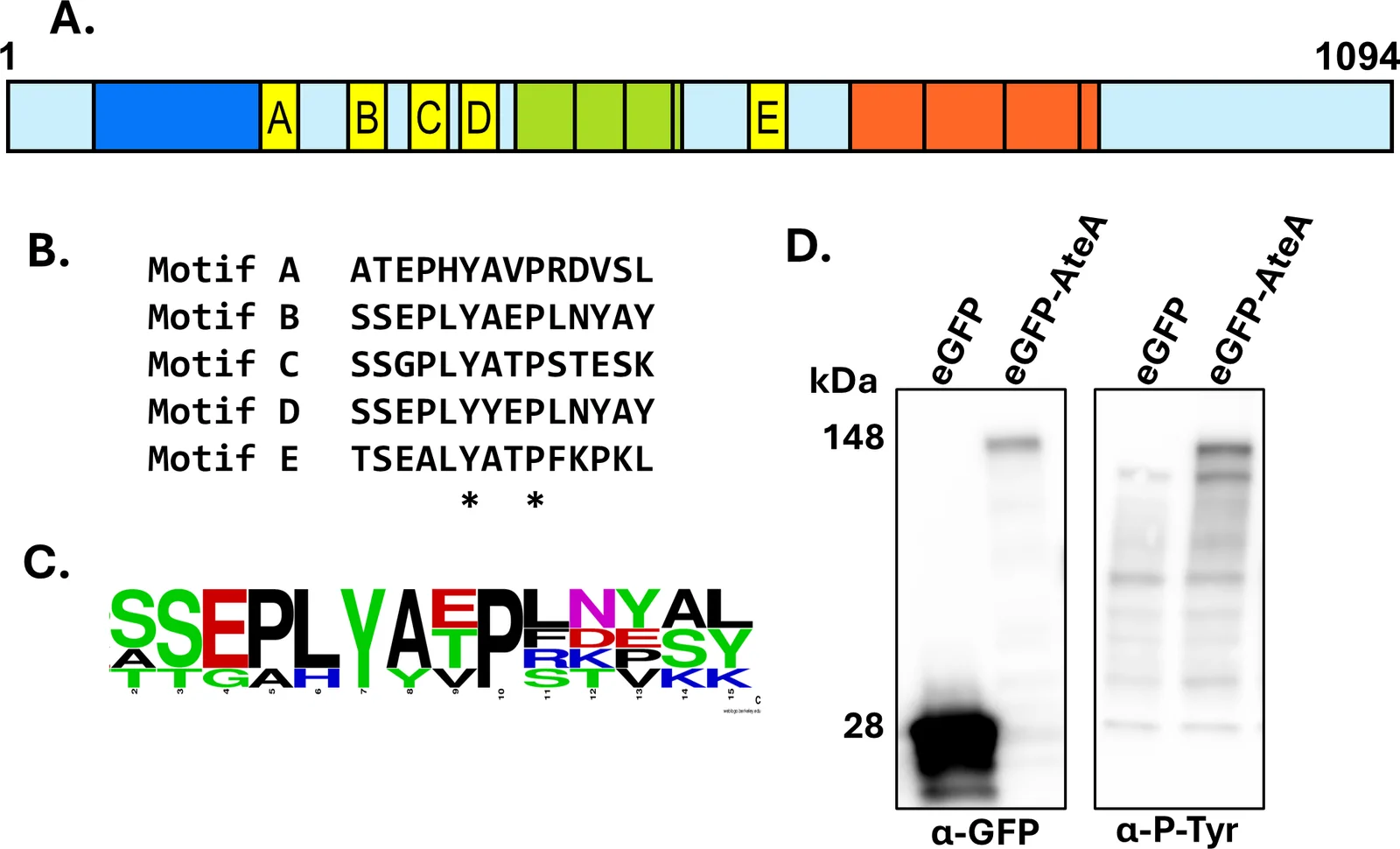
AteA contains EPLYA/EPIYA-like motifs and is tyrosine-phosphorylated in eukaryotic cells. (**A**) Schematic diagram of AteA showing the positions of five EPIYA-like motifs (A–E). Dark blue represents the predicted N-terminal globular domain. Green and orange represent the repeat regions. (**B**) The amino acid sequence of the EPIYA-like motifs A–E with flanking sequence. Conserved tyrosine and proline residues are indicated by an asterisk. (**C**) Sequence logo depicting the usage of amino acids in each position of the motif, showing a consensus for EPLYA. (**D**) Western blot analysis of eGFP or eGFP-AteA expressed in HEK293T cells. eGFP-AteA migrates at ~148 kDa and shows robust tyrosine phosphorylation compared to the eGFP control (~28 kDa). The blot is representative of >3 experimental repeats.

Examination of the AteA protein sequence for SLiMs revealed five EPIYA-like motifs (57), which are known to mimic host SH2 domain-binding sites (45–47). Alignment of the five motifs (A–E) (Fig. 1A and B) confirmed EPLYA as the consensus sequence, with all instances of the motif followed by a conserved proline in the +3 position relative to the tyrosine (Fig. 1B and C). This proline at +3 suggests specificity for SH2 domains of signaling proteins Crk, RasGAP, Nck, and/or Vav3 (58–62). Homologs of these proteins are predicted in the *I. scapularis* proteome, posing plausible AteA targets during tick cell infection.

### AteA is phosphorylated at tyrosine residues

For EPLYA motifs to function as SH2-binding sites, they must first be phosphorylated by a host kinase at the tyrosine residue (46). Due to the low transfection efficiency of *I. scapularis* ISE6 cells, HEK293T cells were used as a surrogate system to investigate AteA tyrosine phosphorylation. GFP-tagged AteA was ectopically expressed in HEK293T cells and analyzed by immunoblotting. Anti-GFP blotting confirmed expression of AteA, and anti-phosphotyrosine antibodies showed a strong phosphotyrosine signal corresponding to the ~148 kDa GFP-AteA protein (Fig. 1D). No phosphotyrosine band was observed corresponding to GFP alone. To validate these findings, transfected cell lysates were subjected to immunoprecipitation (IP) using magnetic beads coupled with an α-phosphotyrosine antibody (α-pY), and the immunoprecipitates were analyzed for GFP by western blotting. The α-pY IP captured GFP-AteA but did not enrich GFP alone above background (Fig. S1). Together, these results show that AteA is tyrosine-phosphorylated when in eukaryotic cells.

### AteA is tyrosine phosphorylated during tick cell infection

**Fig 2:**
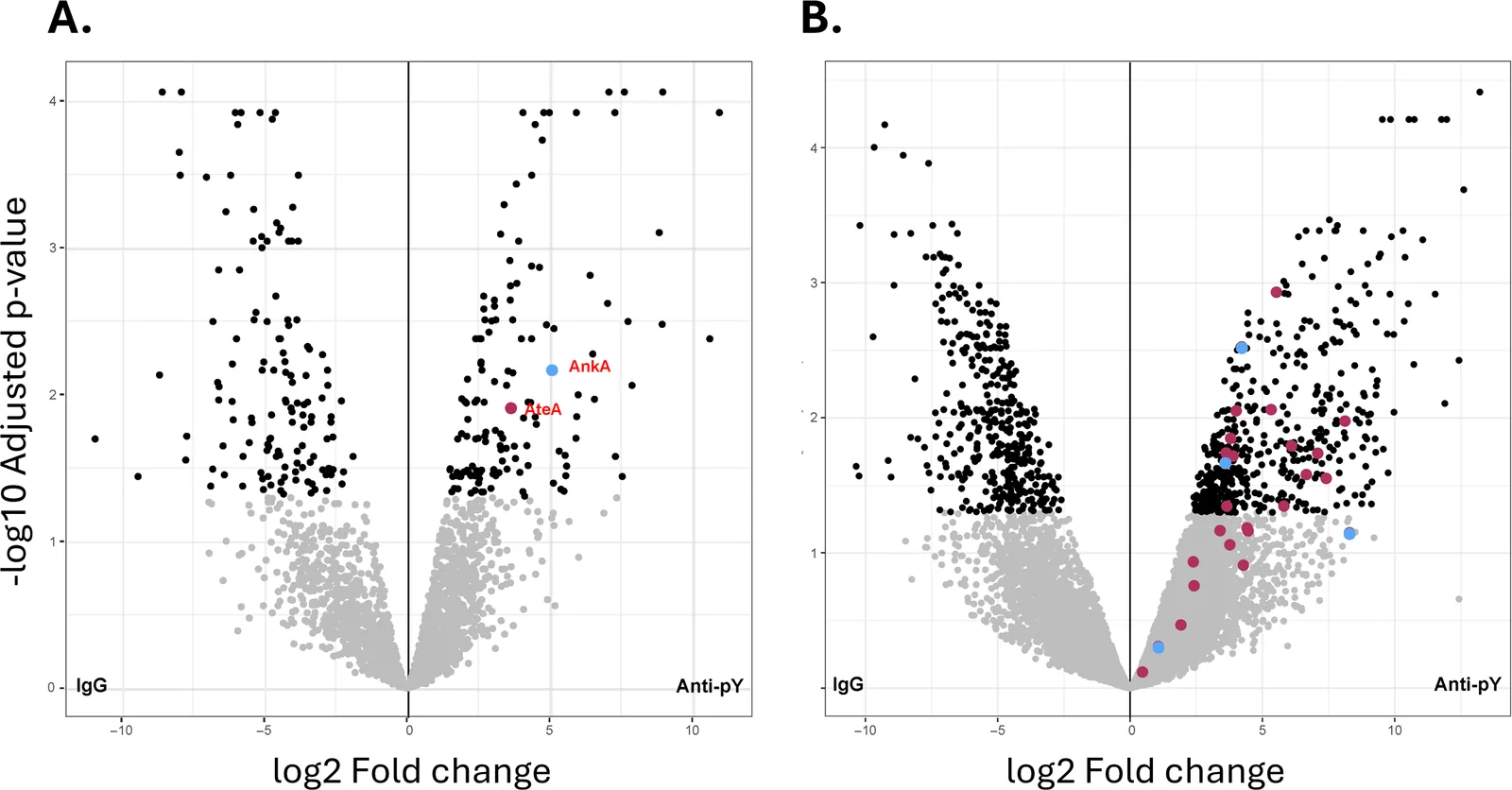
AteA and AnkA are enriched among immunoprecipitated phosphotyrosine proteins. Volcano plots summarizing quantitative comparison of protein immunoprecipitation from lysates of *A. phagocytophilum* infected *I. scapularis* ISE6 tick cells using anti-phosphotyrosine vs non-specific IgG antibodies. (**A**) Depicts all bacterial and tick proteins detected. (**B**) Displays data for each peptide sequence detected. Data points represent the mean of three biological replicates from independent cultures and immunoprecipitations, analyzed sequentially by LC/MS. The plots show −log10 of the Benjamini–Hochberg-adjusted *P*-value (*y*-axis) and log2-transformed relative label-free quantification. Values with *P*-values <0.05 were considered significant (*P*-value < 0.05, black dots; *P*-value > 0.05, gray dots). AteA and AnkA are indicated by magenta and blue, respectively.

Detection of AteA phosphorylation by western blot in *A. phagocytophilum*-infected tick cells is masked by high background of phosphorylated tick proteins. To validate whether AteA is phosphorylated during tick cell infection, infected ISE6 cell lysates were immunoprecipitated (IP) with α-phosphotyrosine antibodies (α-pY) or non-specific rabbit IgG coupled to magnetic beads, and the eluates were submitted for LC-MS/MS analysis (Table S1). In total, 2,549 proteins were detected, including 289 from *A. phagocytophilum*. Among the *Anaplasma* proteins, AteA (WP_021799036.1) was significantly enriched in the α-pY IP (Fig. 2A), represented by 21 detected unique peptides (Fig. 2B). While previous transcriptomics studies reported that expression of the tyrosine-phosphorylated effector gene *ankA* is greatly reduced during tick cell infection, AnkA (WP_020849331.1) was enriched in the α-pY IP but represented by only four unique peptides (Fig. 2B) and at lower abundance than AteA (Table S1). These IP results validate that AteA is tyrosine-phosphorylated during tick cell infection. Further, the detection of AnkA indicates that AnkA is also produced at low levels and phosphorylated during tick infection.

### AteA is phosphorylated at EPLYA motifs

**Fig 3:**
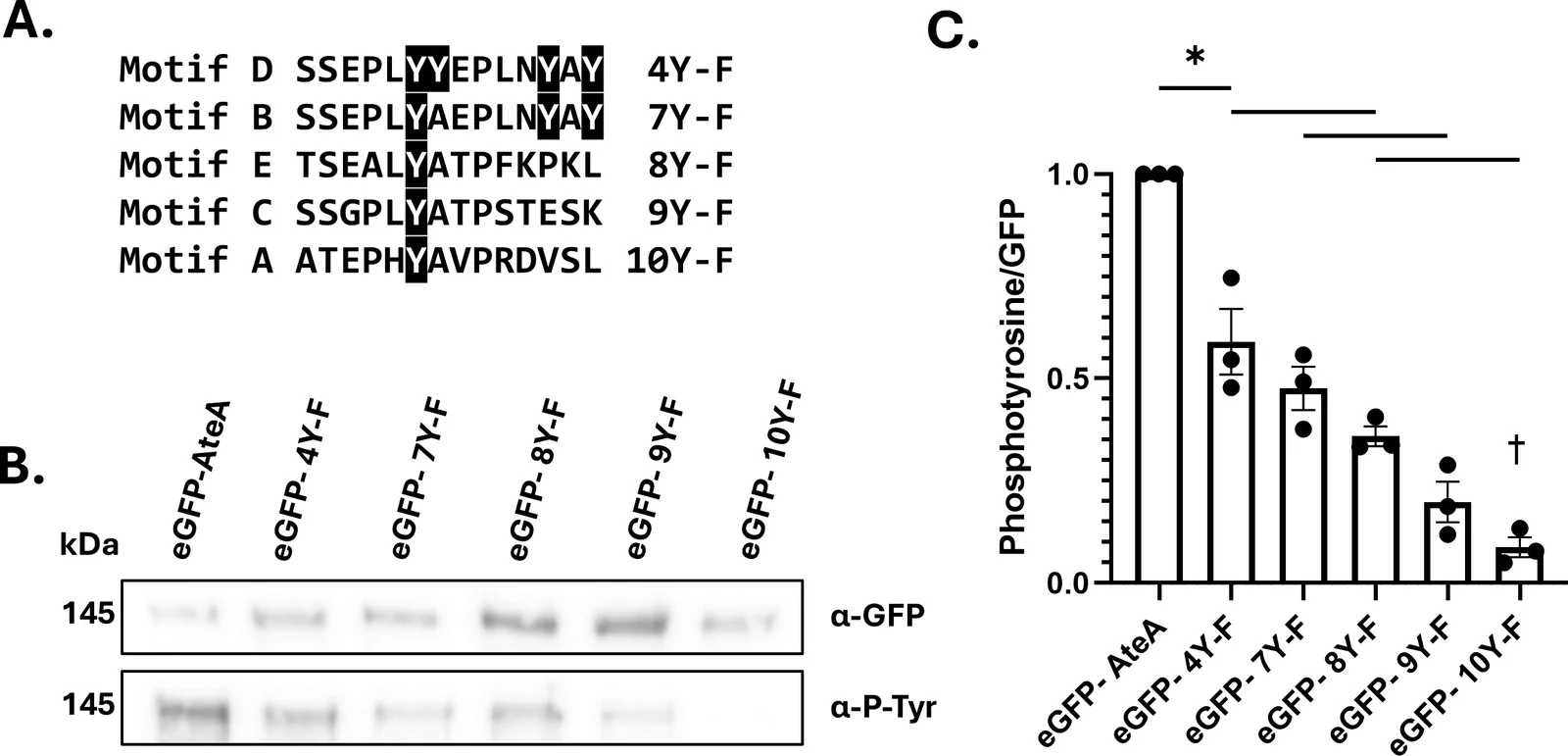
Tyrosine-to-phenylalanine mutations in the EPLYA motifs reduce phosphorylation of AteA. (**A**) Amino acid sequences of the five EPLYA motifs within AteA showing YàF substitutions. (**B**) Western blot analysis of eGFP-AteA and YàF mutant constructs expressed in HEK293T cells. The blot is representative of three experiments. (**C**) Quantification of phosphotyrosine signal normalized to total GFP. Data points represent individual experiments, and bars indicate the mean ± SEM. Statistical significance was assessed by one-way ANOVA (*P < 0.05, †not significant from GFP alone).

To determine if AteA is phosphorylated at EPLYA motifs, site-directed mutagenesis was used to systematically substitute tyrosine (Y) with phenylalanine (F) (Fig. 3A). Phenylalanine was chosen because it closely resembles tyrosine but lacks the hydroxyl group required for phosphorylation (63, 64). The mutations were made first in motif D with subsequent motifs being mutated in an iterative manner (Fig. 3A). HEK293T cells were transfected with plasmids encoding GFP alone, GFP-tagged AteA, or one of the GFP-tagged AteA Y → F variants. Anti-GFP antibodies confirmed protein expression across all variants, while anti-phosphotyrosine antibodies showed a progressive reduction in phosphorylation correlating with the number of Y → F substitutions (Fig. 3B and C). The 4 Y → F mutant exhibited a significant decrease in phosphotyrosine signal relative to wild-type AteA, and the subsequent 7 Y → F to 10 Y → F mutants displayed stepwise decreases in phosphorylation (Fig. 3C). The 10 Y → F mutant showed an almost complete loss of phosphorylation, statistically indistinguishable from the GFP control. These results confirm that the tyrosine residues within the EPLYA motifs are sites of AteA tyrosine phosphorylation and that phosphorylation occurs at multiple sites rather than at a single residue.

### N-terminal region of AteA suppresses tyrosine phosphorylation

**Fig 4:**
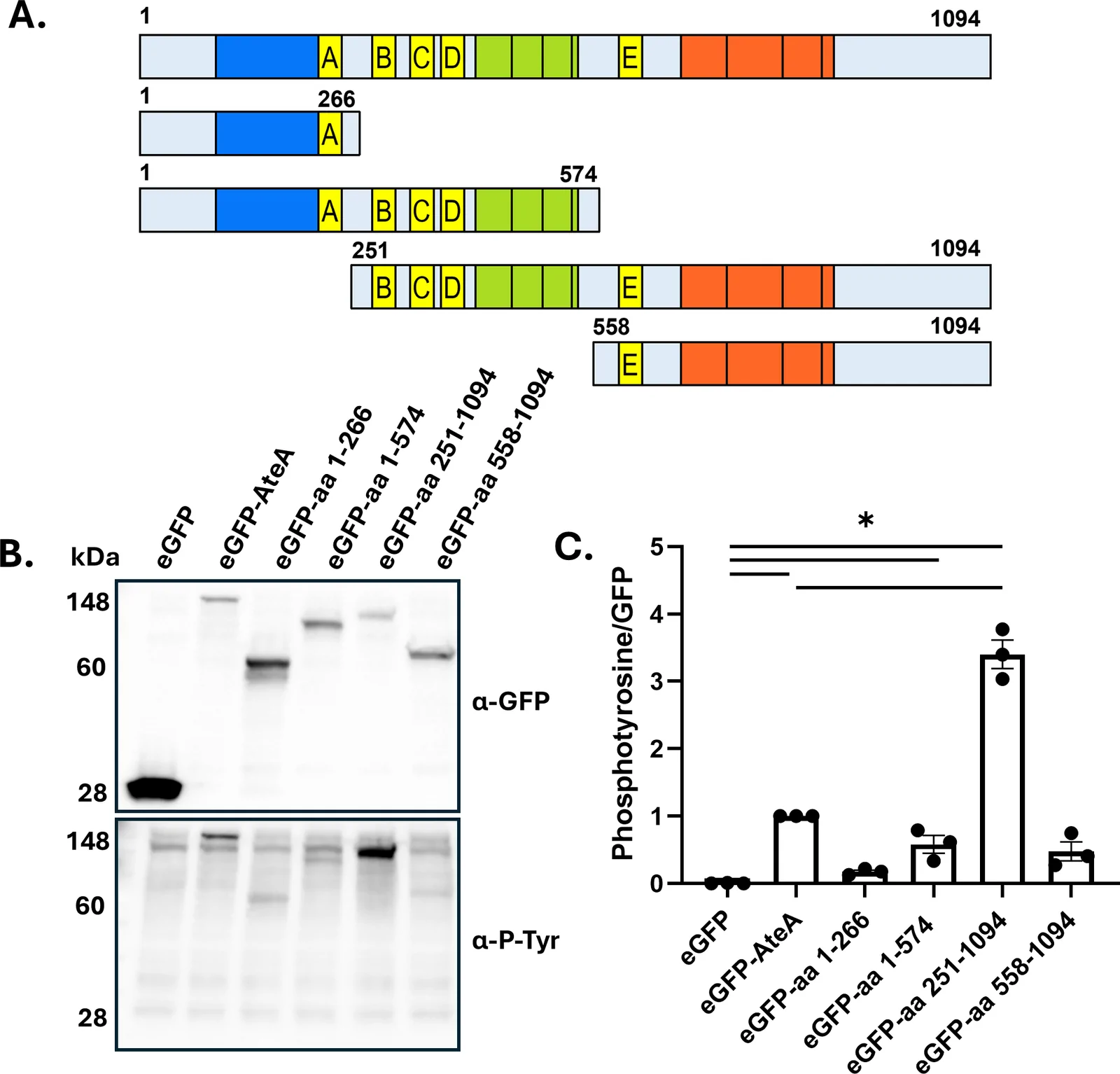
Phosphorylation of AteA truncations expressed in HEK293T cells. (**A**) Schematic diagram of full-length AteA (aa 1–1094) and truncation constructs. The locations of the EPLYA motifs (A–E) are indicated in yellow. (**B**) Western blot analysis of eGFP, eGFP-AteA, and AteA truncation constructs expressed in HEK293T cells. The blots shown are representative of three experiments. (**C**) Quantification of phosphotyrosine signal using all three western blots, normalized to total GFP. Data points represent individual experiments, and bars indicate mean ± SEM. Statistical significance was assessed one-way ANOVA (*P < 0.05).

In our previous study, we showed that multiple regions of AteA influence its localization pattern with actin (22). Therefore, we used these truncation constructs (Fig. 4A) to determine whether tyrosine phosphorylation of AteA EPLYA motifs is impacted by specific regions of AteA or requires the full-length protein. Truncation mutants and full-length AteA (aa 1–1094) were expressed in HEK293T cells and examined for phosphorylation by western blot (Fig. 4B). Blotting with anti-GFP antibodies confirmed detectable expression of all constructs. Phosphotyrosine bands were visible for fragments aa 1–266 and aa 558–1094, each containing only a single EPLYA motif. However, densitometry measurements were not statistically different from background signal in the GFP only control. Phosphorylation of fragment aa 1–574 appeared slightly less than full-length AteA, but was not statistically different by densitometry. Surprisingly, the aa 251–1094 fragment showed a 3-fold greater level of phosphorylation relative to full-length AteA despite losing one EPLYA motif (motif A) (Fig. 4C). The increased phosphorylation of the aa 251–1094 AteA fragment suggested that removal of the N-terminal region (aa 1–251) potentially suppresses the phosphorylation rate of EPLYA motifs in full-length AteA.

### AteA is phosphorylated by host kinases Src and Abl

**Fig 5:**
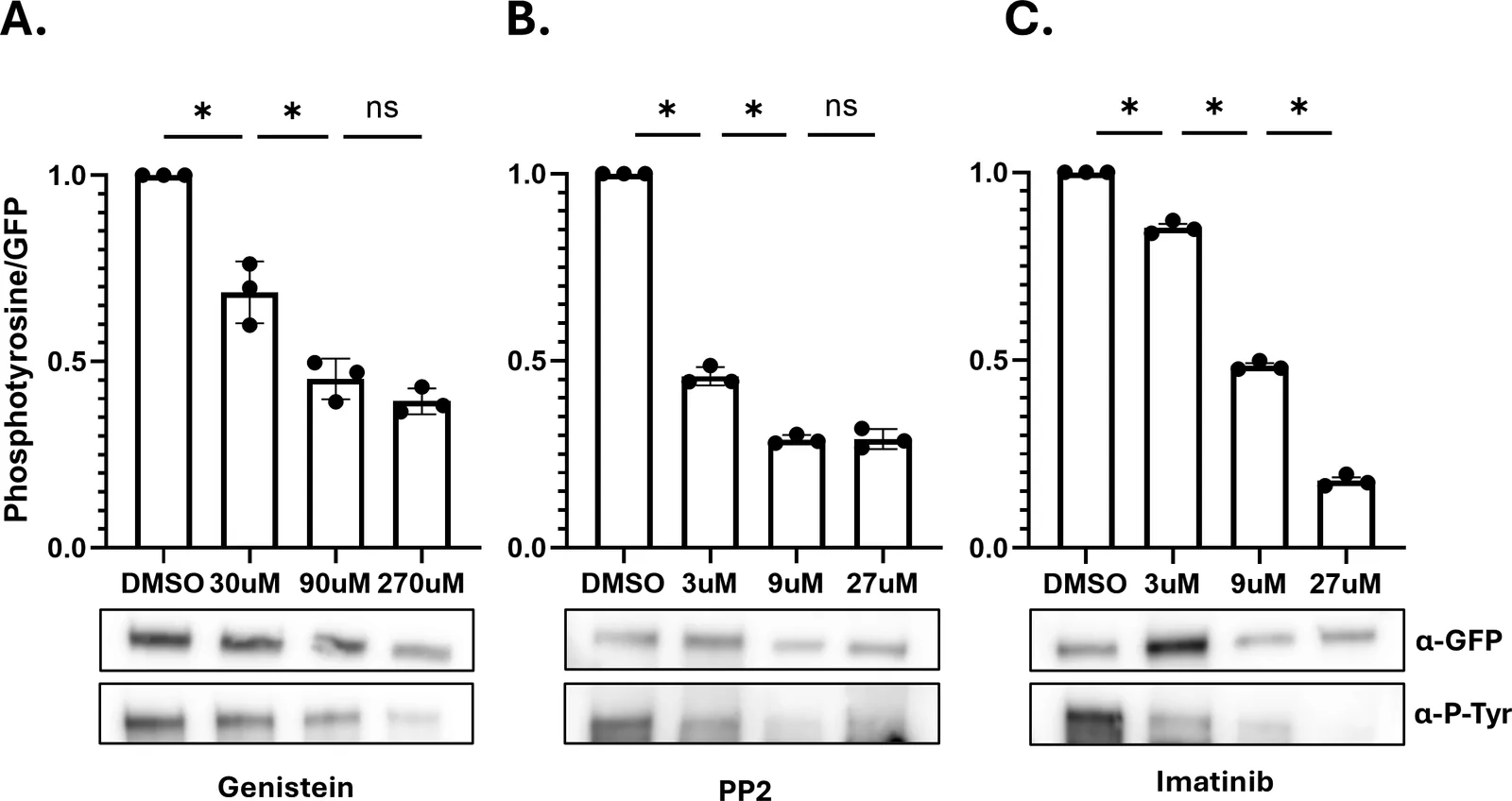
Src and Abl kinase inhibitors reduce AteA phosphorylation. Quantification of phosphotyrosine levels normalized to total GFP following treatment with tyrosine kinase inhibitors genistein (**A**), PP2 (**B**), and imatinib (**C**) at the indicated concentrations, compared with the DMSO control. Corresponding western blots from the same samples show tyrosine phosphorylation detected using anti-phosphotyrosine and total protein level detected with anti-GFP. The blots are representative of three experiments. Data points represent individual experiments, and bars indicate ±SEM. Statistical significance was assessed using one-way ANOVA (*P < 0.05. ns. > 0.05).

Because bacteria lack endogenous tyrosine kinases (65), phosphorylation of AteA must be mediated by host cell kinases. In particular, Src and Abl family kinases are known to phosphorylate EPIYA-like motifs from other bacterial effectors (13, 53, 66), including *A. phagocytophilum* AnkA. To test the role of these kinases in AteA phosphorylation, we used a pharmaceutical approach. The broad-spectrum tyrosine kinase inhibitor genistein served as a positive control, Src family kinases were inhibited using PP2, and Abl kinases were targeted by imatinib. HEK293T cells expressing GFP-tagged AteA were treated with inhibitors at increasing concentrations for 4 h, and protein lysates were subjected to western blotting measuring tyrosine phosphorylation. As expected, genistein caused a dose-dependent decrease in AteA tyrosine phosphorylation (Fig. 5A), confirming that tyrosine kinases are required for AteA modification. Treatment with the Src-family kinase inhibitor PP2 or Abl-family kinase inhibitor imatinib also resulted in a dose-dependent reduction in AteA phosphorylation (Fig. 5B and C), indicating that both Src- and Abl-family host tyrosine kinases contribute to the phosphorylation of AteA and that multiple kinases likely participate in AteA phosphorylation rather than a single dominant kinase.

### Abl and Src are critical for *A. phagocytophilum* tick cell infection

**Fig 6:**
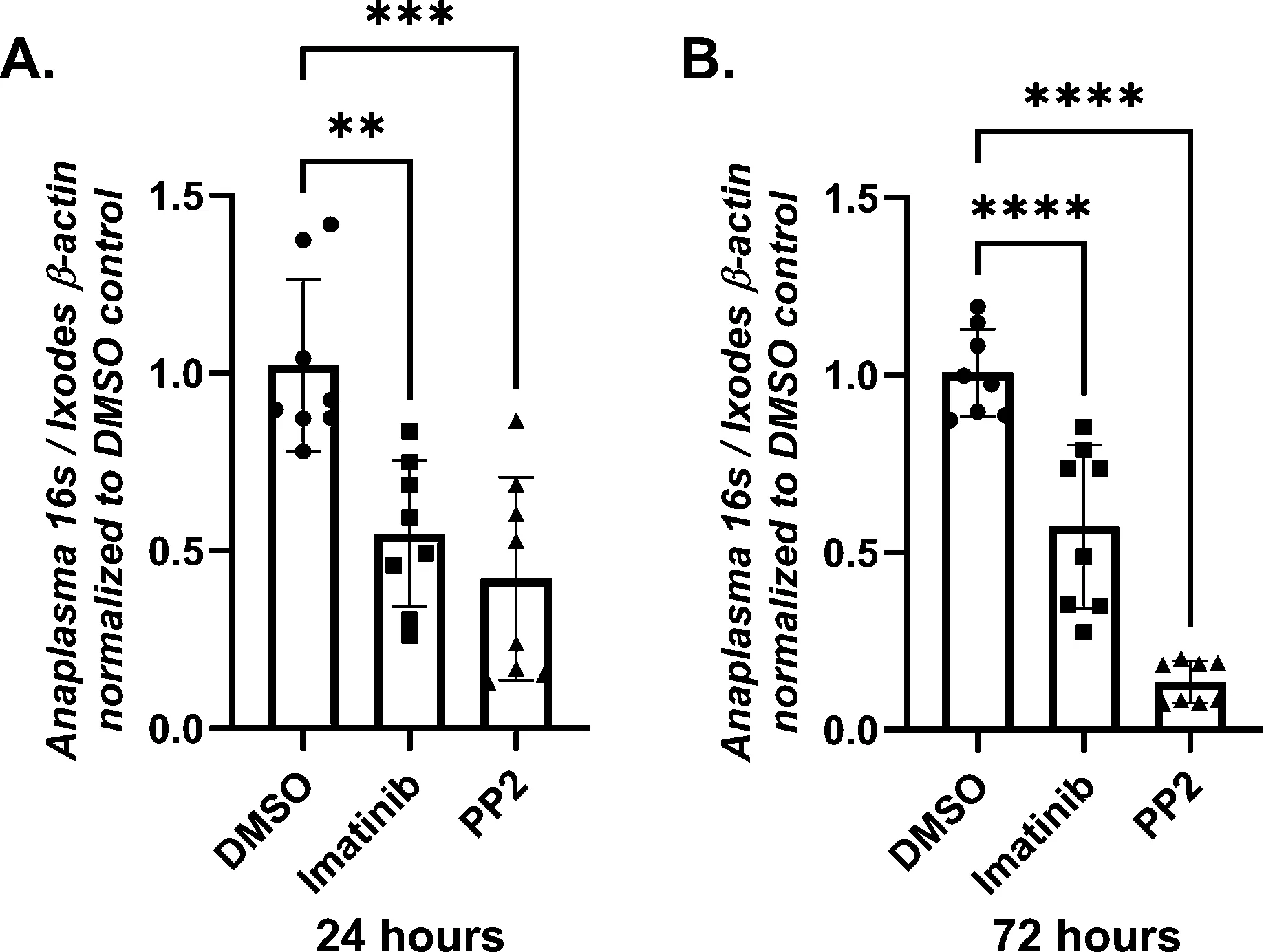
Inhibition of Abl or Src reduces *A. phagocytophilum* during tick cell infection. ISE6 cells were treated with DMSO, imatinib, or PP2 and infected with purified *A. phagocytophilum* bacteria. Bacterial burden was measured at (**A**) 24 and (**B**) 72 h post-infection by qRT-PCR targeting *A. phagocytophilum 16S* vs *I. scapularis β-actin* transcripts. Bar graphs show the mean across two replicate experiments, each with four biological replicate infections shown as points, and bars indicate ± SD. Statistical significance was assessed by one-way ANOVA (**P < 0.005, ***P < 0.0005, ****P < 0.00005).

Previous studies found that inhibition of Src or Abl kinases by PP2 and imatinib, respectively, impaired *A. phagocytophilum* infection in mammalian cells (13, 67). Similarly, suppression of Src by siRNA in ticks or treatment of tick cells with a less specific competitive Src inhibitor (Cayman Chemical, Src Kinase Inhibitor 1) reduced bacterial survival (68). We tested the impact of PP2 and imatinib on *A. phagocytophilum* infection of ISE6 tick cells. The Abl inhibitor imatinib reduced *A. phagocytophilum* burden by ~45% at 24 h post-infection (Fig. 6A), and the level remained similar at 72 h (Fig. 6B). The Src inhibitor PP2 reduced *A. phagocytophilum* burden by 58% at 24 h (Fig. 6A), with a further reduction to 87% at 72 h (Fig. 6B). These results indicate that activities of both kinases are important early during tick cell infection, and Src remains critical as the infection progresses. These changes in bacterial burden may stem from effects on bacterial invasion, survival, and/or replication. Sorting out the exact impacts of these kinases will require future mechanistic study.

### AteA interacts with host signaling proteins RasGAP and Crk through EPLYA motifs

Phosphotyrosine effector motifs, such as the EPLYA motif, mimic canonical SH2 domain-binding sites to mediate interactions with host SH2 domain-containing proteins (46). To evaluate whether the EPLYA and EPLYA-like motifs from AteA can interact with tick SH2 domain proteins, a pull-down assay was performed using commercially synthesized biotinylated peptides that were phosphorylated on tyrosine residues and non-phosphorylated versions of the same peptide (Table 1). Peptides were chosen corresponding to motifs A, B, and C (Fig. 1). Motifs A and C were selected to test two variants of single EPLYA-like motifs while retaining the characteristic conserved proline. To test binding to the double EPLYA motifs (B and D), motif B was chosen because the tandem tyrosines in motif D would complicate the synthesis of the phosphorylated peptide. The bait peptides were individually immobilized on streptavidin beads and incubated with ISE6 whole-cell lysate. Eluted proteins were analyzed by quantitative label-free proteomics. Results were compared between each pair of peptides: phosphorylated vs the non-phosphorylated counterpart. SH2 domain-containing proteins represented by ≥2 peptides, quantified in all three experimental replicates, and enriched by the phosphorylated version of a bait peptide were considered high-confidence binders of the EPLYA motif bait peptides.

**TABLE 1:** Phosphorylated AteA EPLYA motif peptides bind to SH2 domain containing proteins from tick cells^*c*^

Motif peptide^*a*^		Interactor	Protein accession	%^*b*^			#^*d*^			Total abundance (×10^4^)		
				Rep1	Rep2	Rep3	Rep1	Rep2	Rep3	Rep1	Rep2	Rep3
A: SEATEPH[pY]AVPRDVSL		Crk	B7Q2R0	99.9	100	100	8	7	2	2130	580	22.0
		RasGAP	B7P5U8	100	100	100	3	3	3	70.4	64.6	3.73
B: STSSEPL[pY]AEPLN[pY]AYHPIPFK		RasGAP	B7P5U8	100	100	100	7	6	21	399	365	2,530
C: RASSGPL[pY]ATPSTESK		N/A	N/A^*e*^									

^*a*^ Peptides A, B, and C correspond to AteA EPLYA motifs A, B, and C (see Fig. 1).

^*b*^ Percent pulled down with the phosphorylated peptide vs the unmodified peptide.

^*c*^ Reps 1–3 indicate experimental replicates from independent cultures, protein pull-down, and mass spectrometry runs.

^*d*^ Number of peptides identified corresponding to the interactor protein.

^*e*^ NA, no interactors detected.

For motif A, the adaptor protein Crk (B7Q2R0) was the most abundant SH2 protein interacting with the phosphorylated bait peptide, followed by a less abundant interactor, Ras GTPase-activating protein (RasGAP; B7P5U8) (Table 1). For motif B, RasGAP (B7P5U8) was the most abundant interactor and exclusive to the phosphorylated bait peptide pull-downs. In contrast, motif C did not enrich any phospho-specific interacting proteins. Together, these pull-downs identify Crk and RasGAP as candidate AteA interactors.

**Fig 7:**
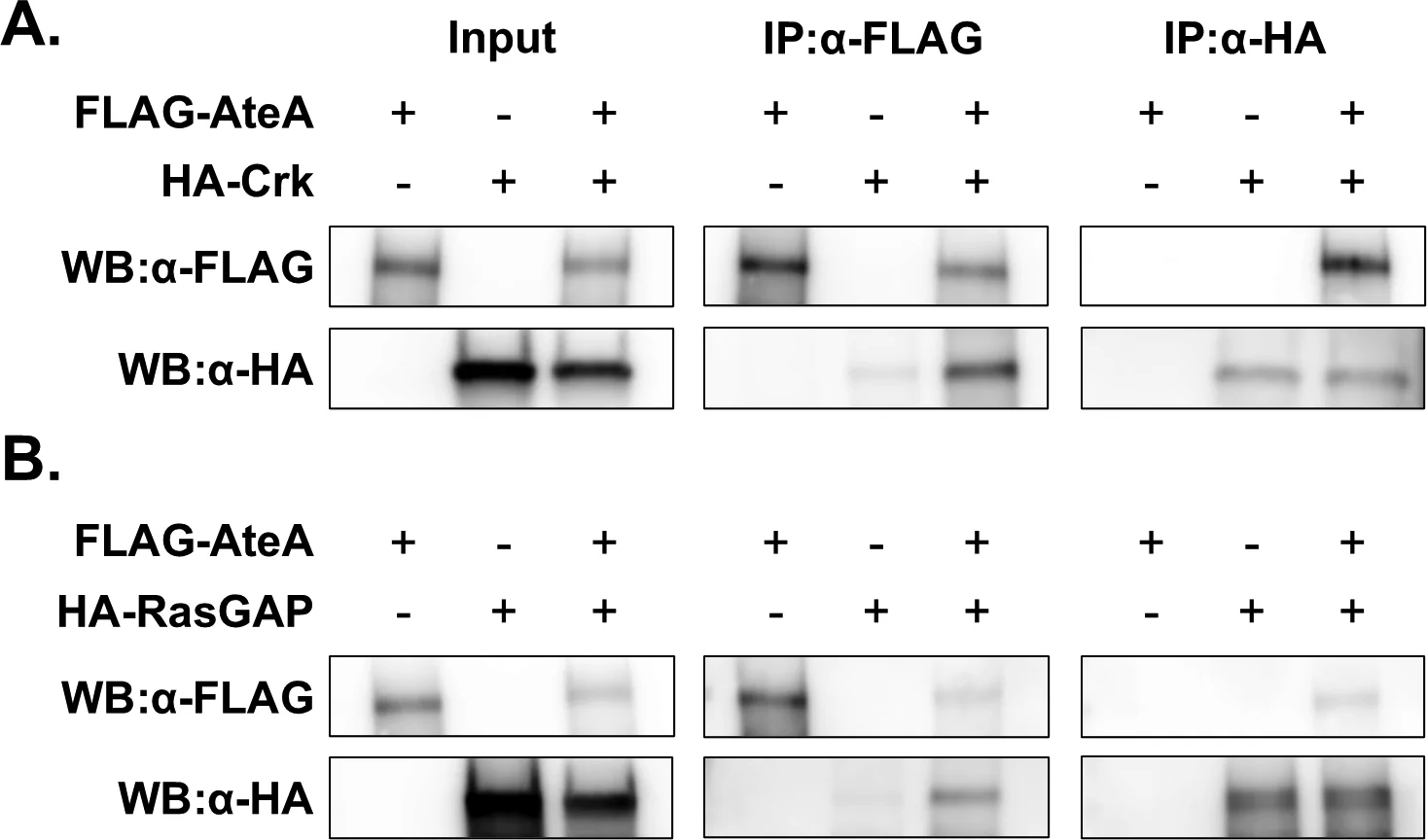
Full-length AteA co-purifies with *Ixodes* Crk and RasGAP. Reciprocal pull-down assay of 3×FLAG-tagged AteA with either HA-tagged *Ixodes* (**A**) Crk or (**B**) Ras-GAP. Recombinant proteins were expressed in HEK293T cells, and cell lysates were used as input for immunoprecipitation. Equal amounts of each sample were loaded on duplicate polyacrylamide gels, transferred to PVDF membranes, and probed with the indicated antibodies. Images shown are from the same single assay and representative of three independent experiments.

To validate whether full-length AteA binds *Ixodes* Crk and/or RasGAP, we performed reciprocal pull-down assays using affinity-tagged proteins expressed in HEK 293T cells. Ectopic expression of each protein was confirmed by western blot using antibodies against FLAG (Flag-AteA) or hemagglutinin (HA) tags (HA-Crk and HA-RasGAP). Immunoprecipitation using anti-FLAG pulled down HA-Crk (Fig. 7A) or HA-RasGAP (Fig. 7B) only when co-expressed with FLAG-AteA. Confirming these results, IP of HA-Crk or HA-RasGAP pulled down FLAG-AteA (Fig. 7A and B). These results support a phosphorylation-dependent interaction between the EPLYA motifs of AteA and the SH2 domain-containing tick proteins Crk and RasGAP.

## DISCUSSION

Our prior study identified AteA as a T4SS effector secreted by *A. phagocytophilum* that is critical for infection of the tick and localizes to the eukaryotic cortical actin cytoskeleton (22). In the present study, we showed that AteA possesses several copies of EPLYA motifs, which were phosphorylated at the tyrosine residue by Src and Abl family kinases. This phosphorylation appears to be dampened by the N-terminal region of AteA through an unknown mechanism. We demonstrated that phosphorylated EPLYA motifs preferentially bind to *I. scapularis* phospho-signaling proteins RasGAP and Crk. Uncovering the mechanisms by which AteA impacts host cell signaling through these interactions is key to understanding how this pathogen survives in the tick vector.

Due to the limited genetic tractability of *A. phagocytophilum*, translocation of T4SS effectors has primarily been tested using heterologous assays. Our study confirmed that AteA is translocated into the tick cell cytosol during infection, as tyrosine-phosphorylated AteA was IP from infected cells. Bacteria lack tyrosine kinases; thus, this phosphorylation was accomplished by host cell tyrosine kinases. The α-pTyr IP identified another *A. phagocytophilum* effector, AnkA, which also contains multiple EPIYA-like sequences (12, 13). AnkA has been primarily examined during mammalian cell infection (14, 15). Although transcriptionally downregulated during tick cell infection (37, 38), detection of AnkA in our data indicates that a small amount of AnkA is present, translocated to the host cell, and phosphorylated during tick cell infection. These two EPIYA-containing effectors that function during tick cell infection suggest that *A. phagocytophilum* leverages these motifs to intervene in tick biology.

Non-receptor tyrosine kinases (NRTKs), including Src family kinases (SFK) and Abelson kinases (Abl), are responsible for phosphorylation of EPIYA motif effectors from several human bacterial pathogens (46, 47), including *A. phagocytophilum* AnkA (12, 13). We found that inhibition of either Abl or SFKs similarly reduced tyrosine phosphorylation of ectopically expressed AteA, suggesting that they both contribute to EPLYA motif phosphorylation. Testing changes in AteA phosphorylation in infected tick cells is limited by the high background and limited genetic tractability of the system; however, we show that pharmaceutical inhibition of Abl and Src hindered bacterial infection of tick cells. Similarly, a previous study showed that siRNA inhibition of tick Src greatly reduced *Anaplasma* survival in tick cell culture and infected ticks (68). Interestingly, the same study found that *A. phagocytophilum* infection affected Src expression differently between fed and unfed ticks (68). Whether the effects of these kinases on tick infection are mediated through AteA phosphorylation has not yet been tested. Abl and SFK have multiple targets within the cell that may also affect the infection (67, 69–71). The relationship between *A. phagocytophilum* and these kinases may extend beyond effector phosphorylation.

Our previous study found that the N-terminus of AteA was responsible for localization to the cell cortex (22). Here, we found that removing this region greatly increased tyrosine phosphorylation. Removing the N-terminus may increase accessibility of the EPLYA motifs to kinases or, conversely, alter their accessibility to phosphatases. Furthermore, the effect on localization could change what resident kinases/phosphatases are in proximity to the AteA protein. The exact interactions mediated by this region remain unknown. Future studies to uncover the role of this N-terminal region will be critical to understand how AteA functions.

SH2 domains generally recognize and bind to phosphorylated tyrosine residues; however, the context of the phosphorylated tyrosine provides specificity for SH2 domain interactions with the residues surrounding the phosphotyrosine, especially the four residues following the tyrosine, providing specificity for different SH2 domain-containing proteins (61, 62). These specificities hold true for bacterial EPIYA effector motifs (46, 47, 49, 72). Protein pull-down assays with AteA EPLYA motif peptides identified *Ixodes* Crk and RasGAP as phosphotyrosine-specific interactors. Both have been previously implicated as targets of other pathogens’ EPIYA effectors (72). CagA from *Helicobacter pylori*, Tir from enteropathogenic *Escherichia coli* (EPEC), and BepF from *Bartonella henselae* have been shown to interact with mammalian RasGAP and Crk (50, 56, 72, 73). While both CagA and Tir also bind to a broader range of SH2 proteins (46, 72), the BepF motif is specific to RasGAP and Crk (72). Both CagA and Tir have a valine at the +3 position after the tyrosine, whereas both BepF and AteA have a proline, likely providing the narrower specificity (61, 62, 72). The exact roles of the RasGAP and Crk interactions with BepF are yet to be determined, but this similarity suggests that AteA and BepF may share a common point of intervention into host cell signaling pathways.

The Erk pathway is a central signaling cascade that broadly regulates cell growth and survival (70). RasGAP functions as a negative regulator of Erk1/2 signaling by stimulating the GTPase activity of Ras, thereby inactivating Ras and the downstream Erk pathway (70). Binding by AteA may inhibit or sequester RasGAP, leaving Erk signaling constitutively active. This could promote cell survival during intracellular growth. In *H. pylori*, the effector CagA activates the Erk pathway through EPIYA interactions with Grb2. CagA can also interact with RasGAP (72), but whether this also contributes to Erk activation is not understood. *Anaplasma phagocytophilum* is known to activate Erk upon invasion of mammalian cells (67, 74). However, AteA expression is specific to tick cell infection (22), where Erk activation has not been examined. Understanding how *A. phagocytophilum* and AteA impact tick cell Erk signaling will require further mechanistic study.

Crk is an adapter molecule that bridges interactions between tyrosine-phosphorylated proteins and components of multiple signaling pathways (73). In particular, Crk proteins play a central role in regulating actin cytoskeleton remodeling (51), making it a critical host determinant of bacterial attachment, host cell movement, and intracellular invasion (50, 56, 73, 75). In our previous work, we found that ectopically expressed AteA localizes to the cortical actin cytoskeleton (22). It is tempting to hypothesize that *A. phagocytophilum* may use AteA interaction with Crk to remodel host cell actin. In *Bartonella,* effector BepF contributes to cell invasion through actin rearrangement (76) and binds Crk through its EPLYA motif. However, removal of the BepF phosphotyrosine residues did not affect invasion rates, raising the possibility that the Crk-BepF interactions are independent of actin rearrangement (77). If and how the cortical actin localization of AteA relates to its interaction with Crk remains to be tested.

It is increasingly understood that ticks actively recognize and respond to *A. phagocytophilum* infection through multiple innate immune and cellular stress response signaling pathways (34, 35, 78–81). While antimicrobial responses mediated by these pathways impact overall bacterial burden, the responses are tempered, leaving the tick competent to harbor and transmit the pathogen (82–85). AteA EPLYA interactors Crk and RasGAP each participate in signaling pathways interconnected with immune response regulation (70, 86, 87). Through these connections, AteA may moderate tick cell responses, enabling *A. phagocytophilum* to persist in the tick.

All together, we found that AteA uses its multiple phosphorylated EPLYA effector motifs to bind tick cell signaling proteins Crk and RasGAP. While a growing number of *A. phagocytophilum* effector interactions have been identified during mammalian cell infection, these findings are the first effector-mediated vector-pathogen protein interactions identified in the tick cell. Uncovering how *A. phagocytophilum* alters tick biology through AteA and its other secreted effectors could inform strategies to disrupt the pathogen in the vector and prevent the transmission to humans.

## MATERIALS AND METHODS

### Bacterial and host cell culture

ISE6 cells derived from embryonic tissues of *I. scapularis* (blacklegged tick) were propagated in L15C-300 medium supplemented with 10% FBS (Avantor, 004E24), 10% tryptone phosphate broth (TPB; BD, B260300), and 0.1% lipoprotein cholesterol concentrate (MP Biomedicals, 219147680). For ISE6 cultures infected with *A. phagocytophilum* HGE1 strain, the medium was further supplemented with 0.25% sodium bicarbonate and 25 mM HEPES buffer (Sigma) as previously described (38, 44). Tick cell cultures were kept at 34°C in an atmosphere containing 4% CO₂. Human promyelocytic cells (HL60; ATCC; CCL-240) were cultured at 37°C with 5% CO_2_ in Roswell Park Memorial Institute (RPMI) 1640 medium supplemented with 10% FBS and 1× GlutaMax. *A. phagocytophilum* HGE1 was grown in HL60 cells, and host cell-free dense core (DC) *A. phagocytophilum* bacteria were isolated as previously described (22, 88, 89).

### Phosphotyrosine immunoprecipitation and proteomics of *A. phagocytophilum* infected ISE6 cells

Three T75 flasks of >90% *A*. *phagocytophilum*-infected ISE6 cells were collected and washed three times with cold PBS containing phosphatase inhibitors (1 mM NaF, 1 mM Na_3_VO_4_, and 0.5 mM sodium β-glycerophosphate). Cell pellets were resuspended in 1 mL lysis buffer (20 mM Tris-HCl [pH 7.5], 150 mM NaCl, 1 mM EDTA, 1% Triton X-100, ProBlock Gold Mammalian Protease Inhibitor, Simple Stop 2 Phosphatase Inhibitor Cocktail, and phosphatase inhibitors as previously described). The cell suspension was sonicated five cycles of 10 s on/10 s off at an amplitude of 30% on ice. Cell debris was pelleted by centrifugation at 14,000 *× g* for 10 min at 4°C. Supernatant was collected, and the protein concentration was determined using the Bradford assay (90).

Three milligrams of total protein was incubated with 20 µL of either Phospho-Tyrosine (P-Tyr-1000) MultiMab Rabbit Monoclonal Antibody Bead Mix (Magnetic Bead Conjugate; #14017; Cell Signaling) or negative control beads conjugated with random IgG Rabbit (DA1E) mAb IgG XP Isotype Control (Magnetic Bead Conjugate; #8726; Cell Signaling) overnight at 4°C with rotation. The beads were washed three times with lysis buffer and six times with 20 mM Tris-HCl (pH 7.5) and submitted for LC-MS/MS analysis at the Molecular Instrumentation Center, University of California, Los Angeles.

On-bead digestion was performed with 0.01 µg/µL trypsin dissolved in 100 mM ammonium bicarbonate at 37°C. Peptides were reduced using 10 mM dithiothreitol (DTT) at 56°C for 1 h and alkylated by adding 40 mM iodoacetamide at RT for 30 min in the dark. The reaction was then quenched with DTT. Trypsin (0.01 µg/µL) dissolved in 100 mM ammonium bicarbonate was added to the digests for a second round of digestion for 2 h at 37°C. Samples were desalted using Empore StageTips, dried in a speed vacuum, and resuspended in 3% acetonitrile containing 0.1% formic acid.

Digested peptides were injected into an IonOpticks C18 reverse-phase column (8 cm × 150 µm, 1.7 µm particle size) maintained at 52°C, with electrospray ionization into an Orbitrap Astral mass spectrometer. Chromatographic separation was performed using a trap-and-elute workflow on a Vanquish Neo UHPLC system. Data-independent acquisition (DIA) was performed on the Astral mass spectrometer operating in positive electrospray ionization mode. Results were annotated using DIA-NN software against the *A. phagocytophilum* HGE1 and *I. scapularis* ISE6 cell proteomes (GCF_000478425.1, GCF_002892825.2). Results were quantified, compared, and displayed as volcano plots using FragPipe-Analyst software (91).

### Culture and transfection of HEK-293 cells

HEK-293T cells (a derivative of human embryonic kidney cells) were maintained in Dulbecco’s modified Eagle medium (Cytiva DMEM, HyClone, Logan, UT) supplemented with 5% FBS and incubated at 37°C under humidified conditions with 5% CO_2_. For transfection, cells were seeded at 1 × 10^5^ cells/well in a 6-well plate. At 70–90% confluency, cells were transfected with Lipofectamine 3000 (Life Technologies, Carlsbad, CA, USA), according to the manufacturer’s instructions. Transfected cells were incubated for 48 h at 32°C with 5% CO_2_ to express the proteins. Protein expression was verified by live cell microscopy for GFP fluorescence and/or western blot against protein tags (GFP, FLAG, or HA).

### Phosphotyrosine immunoprecipitation of AteA-transfected cells

Transfected HEK-293T cells expressing eGFP or AteA-eGFP plasmids were washed once with cold 1× PBS containing phosphatase inhibitors (1 mM NaF, 1 mM Na3VO4, and 0.5 mM sodium β-glycerophosphate). Cells were disrupted in lysis buffer (20 mM Tris-HCl [pH 7.5], 150 mM NaCl, 1 mM EDTA, 1% NP-40, ProBlock Gold Mammalian Protease Inhibitor, and Simple Stop 2 Phosphatase Inhibitor Cocktail) and incubated for 5 min on ice, followed by sonication on ice at 30% amplitude for five cycles of 10 s on and 10 s off. Cell lysates were cleared by centrifugation at 14,000 × *g* for 10 min at 4°C. Protein concentrations of lysates were measured by Bradford assay. Equal amounts (500 µg) of cell lysates were used for the immunoprecipitation assay. Ten microliters of Phospho-Tyrosine (P-Tyr-1000) MultiMab Rabbit Monoclonal Antibody Bead Mix (Magnetic Bead Conjugate) (14017; Cell Signaling) was added to the cell lysates and incubated at 4°C overnight with rotation. Next, the beads were washed five times with the lysis buffer (similar composition as the cell lysis buffer, except that 0.1% NP-40 was used instead of 1% NP-40). After the final wash, 50 µL of 3× Laemmli buffer was added and heated at 100°C for 5 min. Tubes were placed onto the magnetic stand, and the elute was collected into a new tube. Protein samples were analyzed by western blot for phosphotyrosine and GFP, described below.

### Western analysis

Proteins were separated using 4–15% Mini-PROTEAN TGX precast gels (Bio-Rad; 4561083). Proteins were transferred to polyvinylidene fluoride (PVDF) membranes and blocked in 5% bovine serum albumin (BSA) in TBS-T (1× Tris-buffered saline + 0.1% Tween-20). Blocked membranes were incubated overnight at 4°C with primary antibodies, including α-phospho-tyrosine (1:2,000) (Cell Signaling Technology, 8954), α-GFP (1:1,000) (rabbit; Cell Signaling Technology, 2956), mouse anti-eGFP (1:1,000) (MA1-952, Invitrogen), α-FLAG antibody (1:2,000) (anti-FLAG M2-peroxidase [HRP], mouse monoclonal; Millipore Sigma, A8592), or α-HA antibody (1:1,000) (HA-Tag [C29F4] rabbit monoclonal antibody; Cell Signaling Technology, 3724). The membranes were washed three times with TBST before incubation with HRP-conjugated goat anti-rabbit IgG secondary antibody (1:3,000) (Cell Signaling Technology; 7074) or goat anti-mouse IgG secondary antibody (1:5,000) (G-21040, Invitrogen) for 2 h at 4°C. Protein bands were visualized using the SuperSignal West Pico PLUS Chemiluminescent Substrate (Thermo Fisher Scientific) and imaged with an Azure 300 Chemiluminescent Imaging System. To reprobe the membranes with a different antibody, membranes were stripped with Restore PLUS Western Blot Stripping Buffer for 20 min at RT.

### Kinase inhibitor treatment of transfected cells

Stocks of genistein (Abcam; ab120112), imatinib mesylate (Abcam; ab142070), and PP2 (Src and RIP2 kinase inhibitor) (Abcam; ab120308) were prepared in DMSO at concentrations of 20 mM, 20 mM, and 10 mM, respectively. HEK293T cells were transfected to express GFP-AteA. Following transfection, proteins were expressed for 48 h at 34°C and 5% CO_2_. Transfected cells were treated with final concentrations of 30 µM, 90 µM, and 270 µM of genistein and 3 µM, 9 µM, and 27 µM of imatinib mesylate and PP2. Control treatments were equivalent volumes of DMSO. Cells were treated with the specified inhibitors for 4 h, harvested, and lysed in 2× Laemmli sample buffer (Bio-Rad; 1610737). Protein samples were analyzed by western blotting for GFP and phosphotyrosine. Protein bands were quantified by densitometry using ImageJ.

### Kinase inhibition during tick cell infection

ISE6 cells were seeded to 24-well plates at 5 × 105 cells/well in infection media. Cells were allowed to adhere to the plate overnight and then were treated with indicated concentrations of PP2, imatinib, or equivalent volumes of DMSO. At 1 h post-treatment, cells were infected with host cell-free *A. phagocytophilum* HGE1 DC cells at an estimated MOI of 30. Cells from four replicate wells were collected at indicated time points and washed twice with cold PBS. Pelleted samples were processed for RNA using Zymogen Quick-RNA MicroPrep Kit, followed by DNAse treatment (Invitrogen TURBO DNA-free Kit). Relative live bacteria burden was quantified by qRT-PCR of *A. phagocytophilum* 16S rRNA relative to *I. scapularis* β*-actin* transcripts, as previously described (22, 89), and compared with that of the DMSO vehicle control using the ΔΔCt method.

### Site-directed mutagenesis

Full-length AteA was amplified from *A. phagocytophilum* (HGE1 strain) genomic DNA and used as the template for site-directed mutagenesis. Tyrosine-to-phenylalanine (Y→F) substitutions were introduced by overlap extension PCR using the primers listed in Table 2. The resulting mutants were cloned into the Gateway pDONR 221 vector (Thermo Fisher Scientific) using BP Clonase (Invitrogen) and subcloned into the Gateway destination vector pDEST-GFP (Addgene) using LR Clonase (Invitrogen) (22). The resulting clones were confirmed by DNA sequencing.

**TABLE 2:** Primers used for cloning and site-directed mutagenesis

Primer name	Primer sequence
AteA F	GGGG ACA AGT TTG TAC AAA AAA GCA GGC TTA ATG GGA AAA TTA ACA AAA ATC
AteA R	GGGG AC CAC TTT GTA CAA GAA AGC TGG GTA CTA GAA ACG TGC CCT
Y→F 405-413 F	CCA CTC TTT GCT GAA CCC TTA AAT TTC GCC TTC CAC CCT ATT CCT TTT AAG AGG CAC AAC
Y→F 405-413 R	ATT TAA GGG TTC AGC AAA GAG TGG TTC GCT ACT AGT ACT ACT ACT TTG AGT AGG GGT ATC
Y→F 316, 322, 324 F	CCA TTG TTC TTC GAG CCA CTA AAC TTT GCA TTT CAT CCT ATT CCT TTT AAG ACC AAC ACT
Y→F 316, 322, 324 R	GTT TAG TGG CTC GAA GAA CAA TGG CTC GCT ACT ACT ACT ACT TGG AGT AGG AGA TTG
Y→F 242 F	CTG AAC CCC ACT TTG CTG TCC CTC G
Y→F 242 R	CGA GGG ACA GCA AAG TGG GGT TCA G
Y→F 366 F	GTA GCG GAC CGT TAT TTG CTA CTC CTT CTA C
Y→F 366 R	GTA GAA GGA GTA GCA AAT AAC GGT CCG CTA C
Y→F 639 F	GCT CAA CTA GCG AAG CAT TAT TTG CTA CTC CTT TTA AAC C
Y→F 639 R	GGT TTA AAA GGA GTA GCA AAT AAT GCT TCG CTA GTT GAG C
Crk-F	TAA GCA GAA TTC ATG AGG GAC TTT CAC ATA ATT TTC G
Crk-R	TAA GTT GCG GCC GCC TAC CTC GCT GTA TCC ATG

### EPLYA peptide pull-down

ISE6 tick cells were cultured in T75 flasks until 80–90% confluency. Adherent cells were washed three times with ice-cold PBS and then lysed in 2 mL of lysis buffer (50 mM Tris-HCl, pH 7.5, 150 mM NaCl, and 1% NP-40) supplemented with ProBlock Gold Mammalian Protease Inhibitor and Simple Stop 2 Phosphatase Inhibitor Cocktail. Cells were scraped, and lysates were sonicated on ice using two cycles of 5 × 30 s ON/OFF at 10% amplitude. Following sonication, lysates were centrifuged at 14,000 × *g* for 10 min at 4°C to pellet debris. Protein concentrations in supernatant were determined using the Bradford assay (90). Four nmol of each biotinylated peptide were incubated with 75 µL of streptavidin magnetic beads (Pierce Streptavidin Magnetic Beads, 88816) for 1 h at RT with gentle rotation. The beads were washed three times with 1 mL of TBS containing 0.05% Tween-20 (TBS-T) and then incubated under rotation overnight at 4°C with 1 mg of total ISE6 cell lysate in the presence of protease and phosphatase inhibitors (ProBlock Gold and Simple Stop 2). The next day, beads were washed three times with 1 mL TBS-T, followed by three washes with TBS.

Protein digestion and LC-MS were performed at the Washington State Tissue Imaging, Metabolomics, and Proteomics Laboratory (TIMPL). Beads were washed three times with 50 mM ammonium bicarbonate and suspended in 100 mM ammonium bicarbonate containing 1 M urea. Proteins were reduced with 20 mM DTT and alkylated with 40 mM iodoacetamide for 30 min at 30°C for each step. Excess iodoacetamide was subsequently quenched with 10 mM DTT. The on-bead digestion was carried out with 0.5 µg of trypsin (Promega V4111) on a rotator at 37°C overnight, followed by additional digestion with 0.25 µg of trypsin for 3 h at 37°C and 300 rpm using an Eppendorf Thermomixer. The peptides were purified using G-Biosciences C18 spin columns (Cat. #786-931) as per the manufacturer’s instructions. Peptide concentrations were determined using Pierce Quantitative Colorimetric Peptide Assay (Thermo Scientific Cat. #23275).

A total of 300 ng of peptides were used for protein sequencing using an Easy-nLC 1000 UHPLC system coupled to a Fusion Orbitrap Tribrid mass spectrometer. The sample was loaded onto the column in trapping mode, with 3 µL injected. An Acclaim PepMap 100 (75 µm × 20 mm, 3 µm particle size; Thermo Scientific Cat. #164535) was used as pre-column, and a Waters Peptide BEH C18 column (75 µm i.d. × 100 mm, 1.7 µm particle size; Waters Corp., Cat. #186007481) was used as the analytical column.

Raw data were processed using Proteome Discoverer v2.2 and searched against the *I. scapularis* proteome (UP000001555_6945, UniProt October 6, 2024) and a common contaminant database (cRAP) using the SEQUEST HT engine. Only proteins with two or more peptides and present in all three replicates were considered for downstream analysis. Interaction partners of phosphor-Y peptides were selected based on exclusivity or significant enrichment in phosphopeptide samples (protein absent in non-phosphorylated peptide).

### Reciprocal pull-down assay

The full-length AteA was subcloned from pDONR 221 into pCAGGS 3×FLAG-tag vector using the Gateway LR Clonase system, placing a 3×FLAG tag at the N-terminus of AteA under the control of the chicken β-actin promoter. Crk was PCR-amplified from ISE6 cDNA using gene-specific primers (Table 1) and cloned at XhoI/NotI sites of the pCMV-N-HA vector, generating the pCMV-N-HA-Crk expression plasmid. A 2,810 bp sequence from *I. scapularis* encoding RasGAP was codon-optimized for expression in mammalian cells and cloned into the EcoRI/NotI sites of the expression vector pcDNA3.1(+)-N-HA, generating the plasmid RasGAP-pcDNA3.1(+)-N-HA (GenScript USA Inc.). Both Crk and Ras-GAP had an HA tag at the N-terminal end and were under the control of the CMV promoter.

HEK293T cells were transfected with either paired or single expression plasmids. Transfected cells were washed twice with 1 mL of ice-cold 1× PBS. Cell lysates were prepared by adding 400 µL of IP-lysis buffer (25 mM Tris-HCl, 150 mM NaCl, 1% NP-40, 1 mM EDTA, 5% glycerol, 1× protease inhibitor cocktail) to each well. The lysates were incubated for 5 min on ice and then sonicated three times for 20 s per pulse at 30% amplitude. Cell lysates were cleared by centrifugation at a maximum speed for 10 min. Protein concentrations were measured using the Quick Start Bradford Dye Reagent (Bio-Rad, Hercules, CA, USA).

Pull-down assays were performed using either anti-FLAG beads (Pierce Anti-DYKDDDDK Magnetic Beads, Thermo Scientific, Rockford, IL) or anti-HA beads (Pierce Anti-HA Magnetic Beads, Thermo Scientific, Rockford, IL, USA) according to the manufacturer’s protocols. The slurry of magnetic beads was added to microcentrifuge tubes and washed twice with IP lysis buffer for anti-FLAG beads and 0.05% TBS-T for anti-HA beads. An equal amount of cell lysates was added onto the washed beads and incubated at 4°C overnight on a rotating platform. The beads were collected with a magnetic stand and washed with the wash buffer at least five times. After the final wash with ultrapure water, the beads were suspended with 100 µL SDS buffer, mixed, and incubated for 10 min at 95–100°C to elute the proteins from the beads. The supernatants containing the pulled-down protein samples were subjected to western blot analysis using antibodies against FLAG- and HA-tagged proteins, as described above.

## SUPPLEMENTAL MATERIAL

Figure S1 mbio.02165-26-s0001.pdf Phosphotyrosine immunoprecipitation captures GFP-AteA but not GFP alone.

Table S1 mbio.02165-26-s0002.xlsx DE results.

ASM does not own the copyrights to Supplemental Material that may be linked to, or accessed through, an article. The authors have granted ASM a non-exclusive, world-wide license to publish the Supplemental Material files. Please contact the corresponding author directly for reuse.
