# Supplementary material for: RasGAP and Crk bind Anaplasma phagocytophilum effector AteA at tyrosine-phosphorylated EPLYA motifs
Source: mBio. 2026 Oct 26;17(11):e02165-26. doi: 10.1128/mbio.02165-26 (PMC13753828; doi:10.1128/mbio.02165-26)
Supplement: Supplementary file 1 [file mbio.02165-26-s0001.pdf]

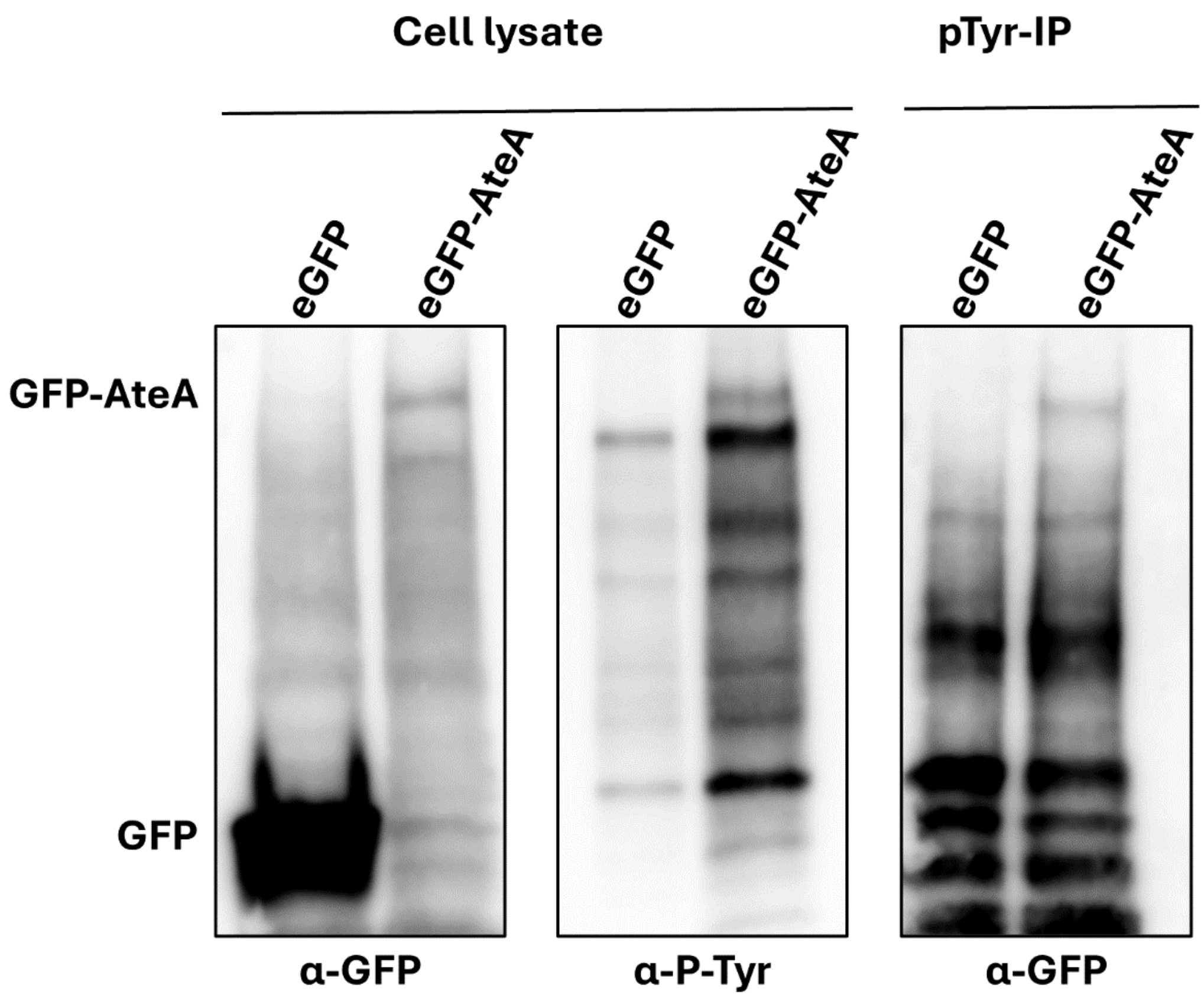

FIG. S1: Phosphotyrosine immunoprecipitation captures GFP-AteA but not GFP alone.

Western blots of Immunoprecipitation (IP) assay of tyrosine phosphorylated proteins from HEK293T cells expressing eGFP or eGFP-AteA. Input lysates and IP elution probed for GFP or phosphotyrosine as indicated. Bands consistent with eGFP-AteA ~148 kDa and eGFP only control (~28 kDa) are indicated. Blots shown are from one replicate of three experiments.
